# Supplementary material for: Light-induced quantum tunnelling current in graphene
Source: Nat Commun. 2025 May 9;16:4335. doi: 10.1038/s41467-025-59675-5 (PMC12064659; doi:10.1038/s41467-025-59675-5)
Supplement: Supplementary file 1 — Supplementary Information [file 41467_2025_59675_MOESM1_ESM.pdf]

Light-induced quantum tunnelling current in graphene

Mohamed Sennary[1], Jalil Shah[1], Mingrui Yuan[1,2], Ahmed Mahjoub[3], Vladimir Pervak[4], Nikolay V. Golubev[1] and Mohammed Th. Hassan[1,2]*.

[1] Department of Physics, University of Arizona, Tucson, AZ 85721, USA.

[2] James C. Wyant College of Optical Sciences, University of Arizona, Tucson, Arizona 85721, USA

[3] Jet Propulsion Laboratory, California Institute of Technology, Pasadena, CA 91109, USA.

[4] Ludwig-Maximilians-Universität München, Am Coulombwall 1, 85748, Garching, Germany.

*Correspondence to: mohammedhassan@arizona.edu

Supplementary Figures

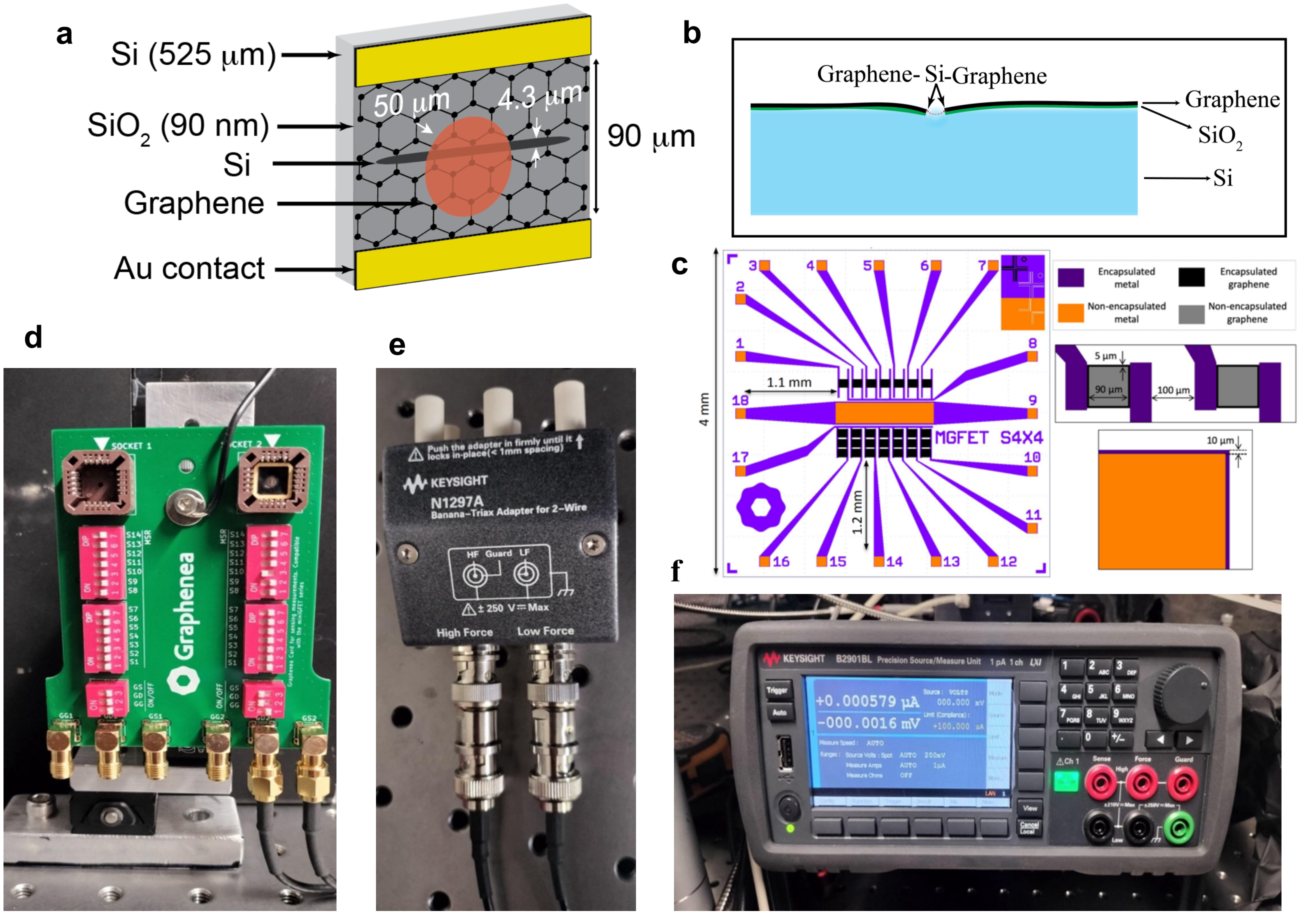

Figure 1: **The phototransistor setup. a**. The graphene channel transistor composition. The illustration shows the Si junction with thickness estimated to be 4.3 μm and the location and the diameter (~50 μm) of the focused laser beam. **b**. cross-section illustration for the junction. **c.** The graphene chip which contain the single- graphene channels and triple-graphene channels sides, adopted from ref [62]. **d**. the two-channels electronic card. **e** the Banana-Triax Adapter (BTA) for 2-Wire (Non-Kelvin) Connection. **f**. the Source / Measure Unit (SMU), Keysight B2901BL model.

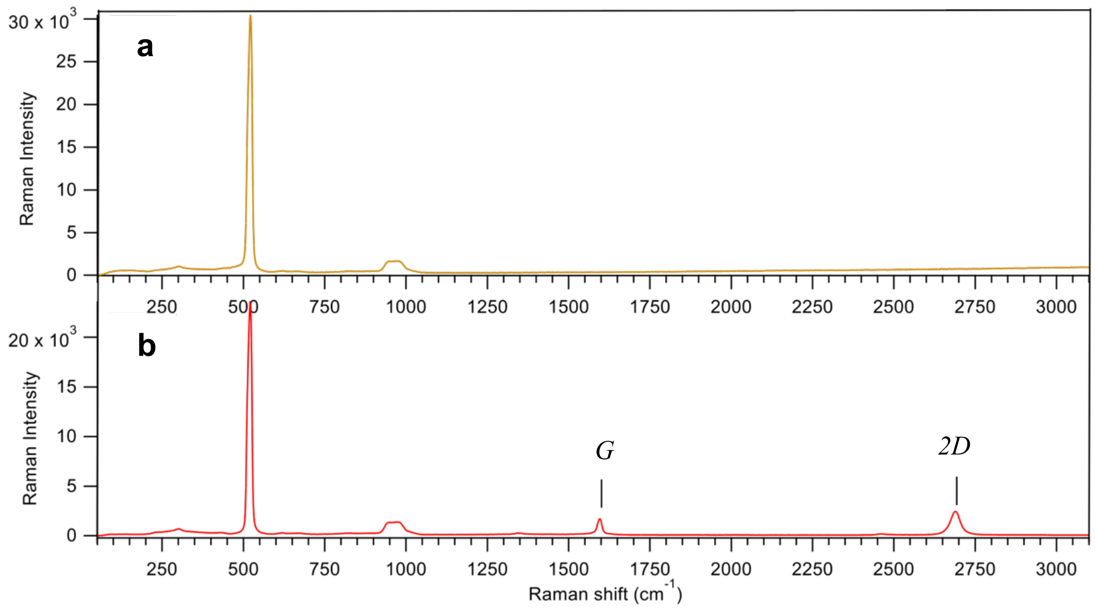

Figure 2: **Raman spectroscopy characterization**. **a**, The obtained Raman spectrum for the junction area in the Gr-Si-Gr transistor. **b**, The Raman spectrum of the reference unmodified graphene channel.

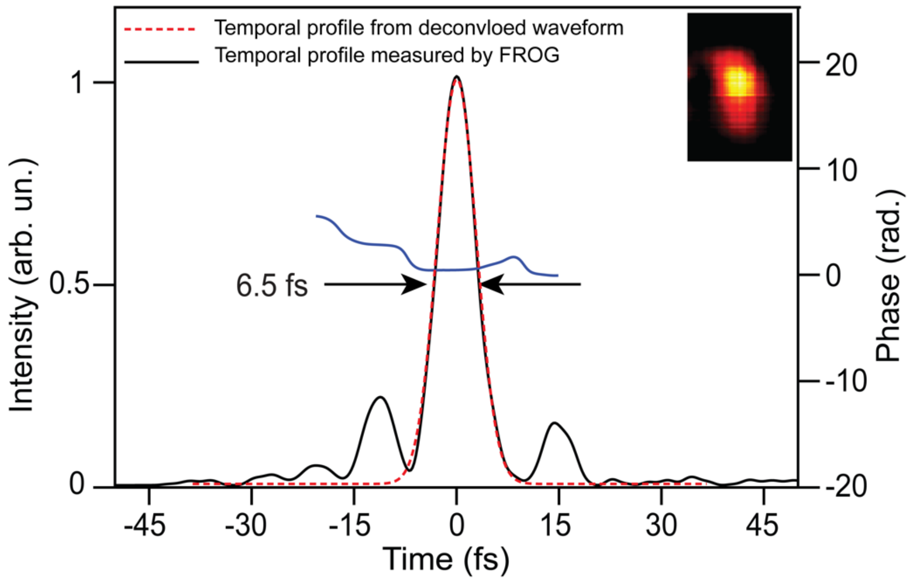

Figure 3: The measured temporal profile of the pump laser pulse retrieved from the FROG measurement (the FROG trace is shown in the inset) is plotted in black line. The spectral phase, plotted in blue colour, indicates that the pulse is almost Fourier limited. The intensity temporal profile of the waveform decomposed from the cross-correlation current measurement is plotted in dashed red line.

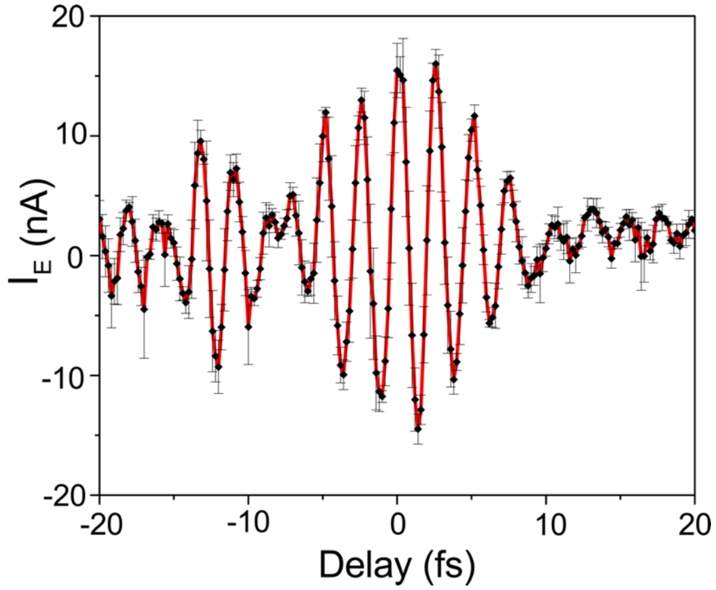

Figure 4. The measured cross-correlation I_E current modulation driven by a field of chirped few-cycle pulse.

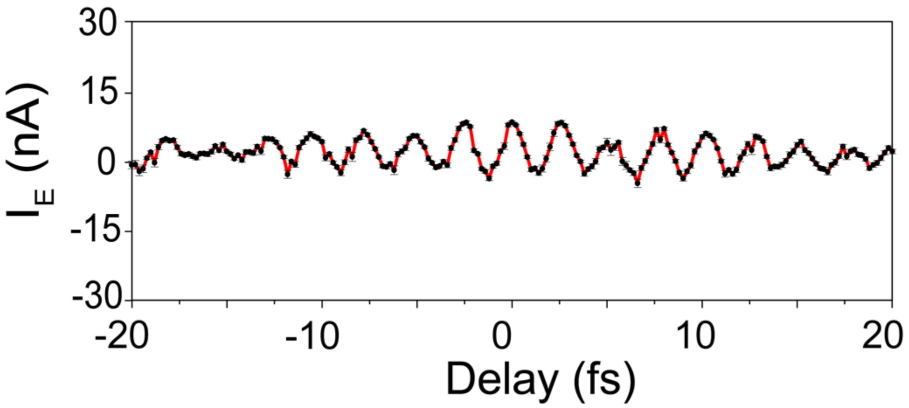

Figure 5: The measured modulated current using circular polarized pulse.

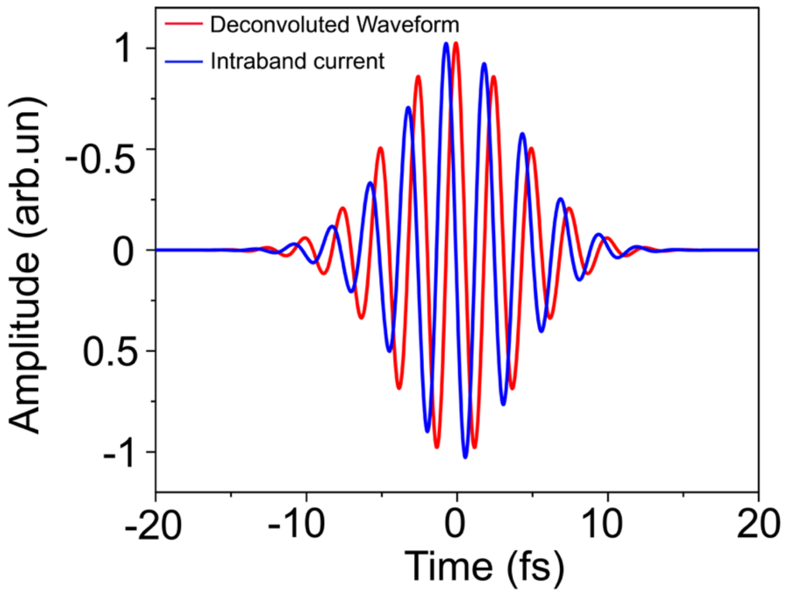

Figure 6. The retrieved deconvoluted waveform of the driver pulse from decomposition of the cross- corelation current measurement is plotted in red line. The intraband current simulated using fully quantum tight-binding model of graphene is depicted in blue line.

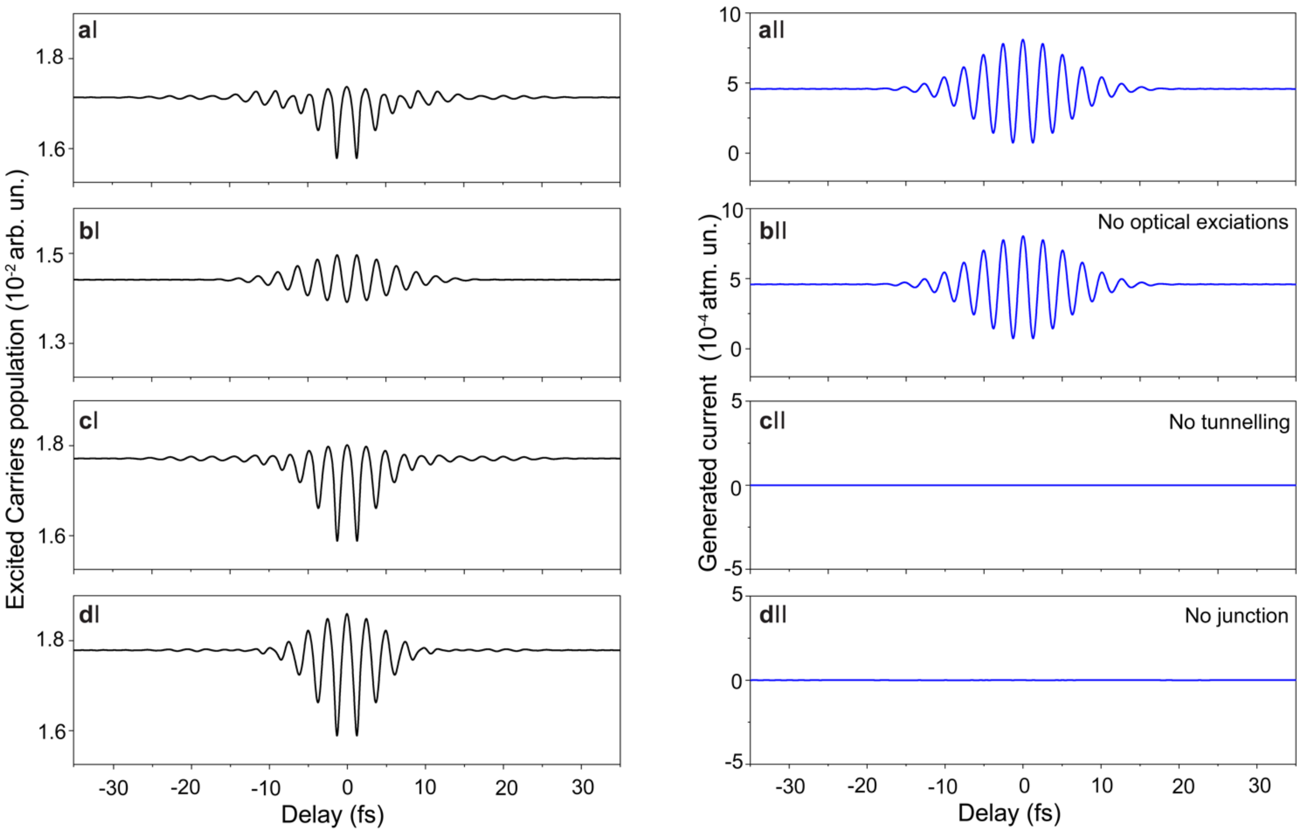

Figure 7. Calculated excited carrier's populations (I) and the residual field-induced generated currents (II) as a function of the time delay between the two laser pulses in Gr-Si junction with (**a**): full account for optical excitations and tunnelling effects, (**b**): optical excitations switched off, (**c**): tunnelling switched off, and (**d**): all effects present but the junction is removed.

Supplementary Notes

Note 1: The phototransistor setup.

The typical graphene device, commercially available from Graphenea®[1], consists of one atomic layer thick graphene channel between the source and drain (see Supplementary Figure 1a). The graphene layer is synthesized using CVD on a SiO_2 (90 nm thick)/Si (525 µm thick) substrate. Each graphene channel is a square of 90 µm side length. The source and drain metallic contacts are Au-based and are encapsulated by aluminium oxide (Al_2O_3) with a thickness of 50 nm. In our experiments, the chip is oriented such that the voltage is applied in the x-axis, and the current is propagating in the same direction accordingly. Each graphene chip has seven single-graphene channel devices and another seven triple-graphene channel devices (connected in parallel) (See Supplementary Figure 1b[62]). Note that all our measurements are taken on the single-graphene channel side.

The SiO_2 junction is formed by a laser-induced depletion process. A focused laser beam with a Gaussian intensity profile is incident on the center of the chip, targeting the multilayer structure of graphene and SiO_2. As the chip is translated along the x-axis, the central, high-intensity portion of the laser beam interacts with the material, causing localized depletion. This depletion results in a arc-shaped region where the graphene and SiO_2 layers are effectively removed (see cross-section illustration in Supplementary Figure 1). The formation of this junction is further supported by Raman spectroscopy scans, as we explain in the next section. Please note that an imaging system was installed to envisage and adjust the laser beam location on the graphene channels. Moreover, the electronic card is mounted on a three linear stages support (See Supplementary Figure 1c), providing access to control the beam size (focus), and location on the chip. Remarkably, the production of this junction is reliable, and the experiments presented in this study have been conducted in different channels, and the results are reproducible. The graphene device is installed on an electronic card (see Supplementary Figure 1c), which consists of a socket that fits the graphene chip, ground connection, 14 source switches for each graphene device, global source, drain, and gate switches. There is one global drain and one global gate for all graphene devices, but each device has its own source connection that is controlled by a switch. The electronic card supports the operation of two chips simultaneously if needed see Supplementary Figure 1c). This card is connected to a precision source/Measure unit (SMU), (Keysight B2901BL) by a N1297A Banana-Triax Adapter (BTA) for a 2-Wire (Non-Kelvin) Connection (See Supplementary Figure

1d&e). The BTA supports low current measurement for less than 1 nA and converts the 4-wire connection into a 2-wire connection in addition to the guard (connected to high force) and ground. Furthermore, the SMU provides a minimum sourcing resolution of 10 pA / 1 μV and a minimum measurement resolution of 1 pA / 100 nV, with a maximum output of 21 V, 1.5 A DC. The SMU is connected to the BTA to have a high force and a low force connection. The high force is connected to the electronic card source, and the low force is connected to the drain. Thus, the SMU provides voltage and measuring current between the source and drain contacts through the graphene channel, while the gate contact has no contribution.

Note 2: Raman spectroscopy characterization

To confirm the composition of the Gr-Si-Gr phototransistor (field-effect transistor device, Fig. 1a right, in the main text), we conducted a Raman spectroscopy characterization. This characterization was conducted using a confocal dispersive Horiba Jobin Yvon LabRam HR Raman spectrometer equipped with a 50 mW, 532 nm (frequency-doubled Nd:YAG) laser. Frequency calibration was executed using a silicon chip, known for its distinctive sharp peak at 520.7 cm^{-1}. Raman spectra were captured at a resolution of 0.5 cm^{-1} per detector pixel, employing a grating with 600 grooves per mm. A 50x objective is used resulting in a laser beam diameter with a high resolution smaller than the junction dark region in the sample (the highlighted region by white rectangular in Fig. 1a right, in the main text). To avoid any change to the sample, we used only 10 % of the laser power, which was enough to record high S/N ratio spectra. Moreover, for reference measurement, we conducted the same characterization on the corresponding area in an unmodified graphene channel. Both obtained spectra of the two samples, shown in Supplementary Figure 2, exhibit a prominent peak at 522 cm^{-1} and a broad peak at 963 cm^{-1}, which can be attributed to silicon material. Furthermore, the spectrum of the unmodified graphene channel sample features distinct peaks at 2690 cm^{-1} (2D peak) and 1596 cm^{-1} (G peak), both characteristic of graphene[2]. The graphene peaks are entirely absent in the spectrum of the junction area in the modified device, indicating a clear displacement of the graphene layer and likely suggesting the removal of the SiO_2 layer, thereby exposing the silicon substrate. Moreover, these measurement shows no indication of graphene oxidization during the conducting experiment since we used a relatively large laser beam diameter at the focus (50 μm), so the intensity is not high enough to induce graphene ionization reaction.

Supplementary References

1. https://www.graphenea.com/

2. Ferrari, A. C. *et al.* Raman spectrum of graphene and graphene layers. *Phys. Rev. Lett.* **97**, 187401, (2006).
