# Supplementary figures and images for: Light-induced quantum tunnelling current in graphene
Source: Nat Commun. 2025 May 9;16:4335. doi: 10.1038/s41467-025-59675-5 (PMC12064659; doi:10.1038/s41467-025-59675-5)

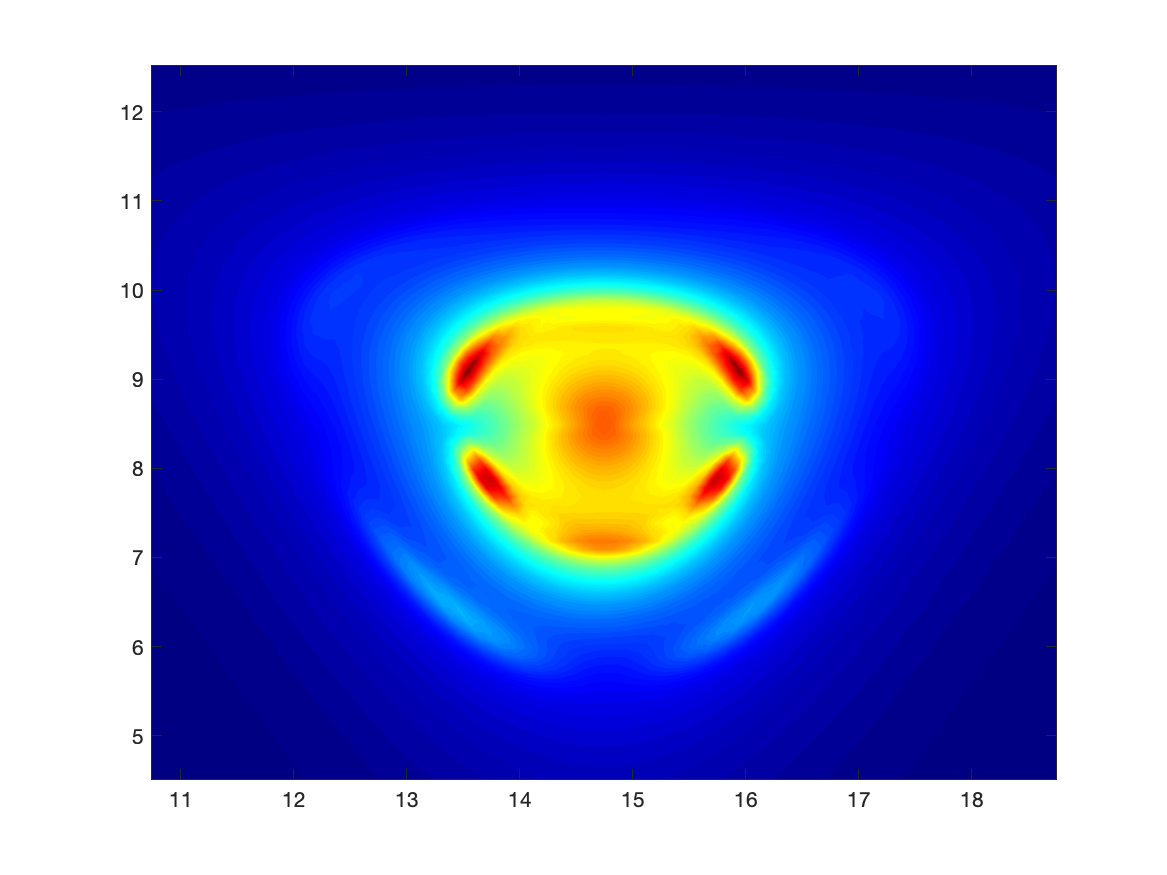

Supplement: Supplementary file 3 — Source Data [file 41467_2025_59675_MOESM3_ESM.zip › Fig.3b/figurex.png]
